# Physiological Effects of Quinia

**Published:** 1877-05-20

**Authors:** 


### PHYSIOLOGICAL EFFECTS OF QUINIA.

Dr. E. L. Drake, of Fayetteville, Tenn., has published in the *Medical and Surgical Reporter* some of his experience and conclusions respecting the physiological action of quinia, which, as they appear to be somewhat different from those commonly attributed to cinchona, may be worth recording in the *Circular*.

He says, at least three different physiological effects can be obtained from this drug. In doses up to two grains it is a tonic ; from this to five grains it is a stimulant, and from this to ten grains or more it is a diaphoretic relaxant and calmative—a febrifuge. The amount of twenty-four grains distributed over twenty-four hours may have some antiperiodic effect in a mild type of ague, but will prove very disagreeable to the patient, on account of chinchonism. If he has any headache, it will aggravate this symptom. If this amount be divided into three doses and taken within four hours, at any time after a pyrexia has reached its height, say after 4 P. M., the toxic effect will be apparent in the relaxation and diaphoresis ; the patient will be drunk. A half pint of whisky can be taken in small doses during a day without inducing intoxication ; yet, if it is all taken in the course of two or three hours, an individual will be apt to exhibit strong evidence of being in this state. So with chloroform and ether. I once treated a pregnant woman who was within a month of term, and suffering from remittent fever, by timidly giving less than five grain doses every three hours ; by the time the third dose was taken uterine pains set in, which soon became expulsive, and forced the child out with a good deal of laceration of the os, although the membranes were not ruptured until the last moment. The contractions were as severe as any I ever saw from ergot, leaving no doubt as to the part the quinine was playing, except as to the provocation. I could not say that it started the pains, yet I am sure that it intensified them in the most fearful manner. In using quinine subsequently with pregnant women, I never gave less than twenty or thirty grains in the space of four or five hours, and never had any unpleasant effects. In malarial regions it is rather common for women to have pains for months before term, which I have found to yleld to large doses of quinine, on the theory that there is a congestion of the womb, and that this agent reduces congestion, especially in the soft and spongy tissues in any part of the body, and in any disease, and does not act antidotally in the usual sense. I make it a rule never to give quinine in the forenoon, always giving it at night, to the amount of not less than twenty-five grains, conjoined with an opiate, and am nearly certain of getting a good diaphoretic effect, which will be apt to extend through the succeeding day. In all the pyrexiæ of this region I prescribe it the first visit, and frequently have the satisfaction of aborting a case of pneumonia, or making its subsequent course manageable. In the pneumonias of the middle-aged, who are so liable to overwhelming congestions of the lungs, it is the sheet anchor, given in large doses at the outset.—*Druggists' Circular*.

